# Supplementary material for: Are carbon emissions trading and green financial instruments synergistic? -Comprehensive quantitative research based on content analysis
Source: PLoS One. 2024 Mar 7;19(3):e0298601. doi: 10.1371/journal.pone.0298601 (PMC10919601; doi:10.1371/journal.pone.0298601)
Supplement: S1 Data — (ZIP) [file pone.0298601.s001.zip › Data/地级市Z_F工作报告.zip!/╣π╢1⁄2/╖≡╔╜╩╨/└┤╫╘╣1⁄2╓┌║┼í╛┬φ┐╦╩2╛▌═°í┐_2009.docx]

各位代表：

　　我代表佛山市人民政府向大会报告政府工作，请予审议，并请政协各位委员和其他列席人员提出意见。

　　一、2008年工作回顾

　　2008年，是国际国内经济形势极为复杂、发展遭遇较大困难的一年。一年来，在省委、省政府和市委的正确领导下，在市人大和市政协的监督支持下，市政府坚持以科学发展观统揽全局，继续解放思想，坚持改革创新，锐意进取，奋力拼搏，一手抓抗击雨雪冰冻、洪涝灾害和支援地震灾区重建家园，一手抓应对国际金融危机，努力推动经济社会全面协调可持续发展，市十三届人大三次会议确定的各项预期目标已经实现。

　　——经济保持平稳较快增长。全市完成生产总值4333.30亿元，增长15.2%；按常住人口计算，人均生产总值72975元（折合10423美元），增长14.3%。地方财政一般预算收入227.99亿元，增长17.19%；税收总额629.41亿元，增长14.94%；

　　全社会固定资产投资1258.65亿元，增长15.5%；社会消费品零售总额1177.84亿元，增长24.4%。

　　——环境质量持续改善。GDP能耗、主要污染物二氧化硫和化学需氧量排放量持续下降，完成预期目标任务。城镇污水处理率达到55%，全市中心城区生活垃圾无害化处理率达到80%。全年空气环境质量达到或优于二级天数为362天。

　　——人民生活水平稳步提高。城镇新增就业岗位21.53万个，城镇登记失业率1.85%。城市居民人均可支配收入22494元，增长9.2%；农村居民人均纯收入9656元，增长7.7%。城乡居民储蓄存款余额3494.11亿元，比年初增长23.1%。

　　一年来，我们主要做了以下几方面工作：

　　（一）以构建现代产业体系为主线，大力推进产业结构调整优化升级

　　现代农业发展成效明显。完成农业总产值198.94亿元，增长1.7％。农业龙头企业发展壮大，新增国家级、省级农业龙头企业各1家。现代农业生态园区建设规划编制完成，新增农业园区面积2.11万亩。海峡两岸农业合作试验区架构初步形成，国通物流成为全国农业保税项目、全国供港澳农产品中转处理中心。农业品牌建设加强，新增农业类广东省名牌产品5个。

　　工业结构调整优化。全社会工业总产值首破万亿元，达11658.31亿元，增长20.0％。大力发展先进制造业，产业高端化逐步显现。全市高新技术产品产值2237亿元，增长6.6%。引入新型显示器件、电子信息、汽车配件、新能源等一批项目。加大传统产业改造提升和落后产能关停并转力度，关停高耗能、高污染、低效益企业622家。积极实施产业和劳动力“双转移”战略，转移企业230家；首期投入资金1亿元，启动佛山（清远）、（云浮）产业转移工业园建设，佛山（清远）产业转移工业园被认定为广东省示范性产业转移园。

　　现代服务业加快发展。金融业发展提速，出台《佛山市金融产业发展规划（2008-2015）》。实施金融发展三项计划[1]，创新试点计划全面展开，创业风险基金、信用担保基金开始运作，企业上市取得突破，新增上市公司5家。广东金融高新技术服务区初具雏形，AIA（美国友邦保险有限公司）后援中心奠基，PICC（中国人民财产保险有限公司）数据中心动工。现代物流业发展加快，成功举办第五届佛山（国际）物流洽谈会，签约项目98项，投资总额263亿元。易运物流市场、三山国际物流园等相继建成运营，“中国-新加坡（佛山）现代物流工业园”项目达成合作意向。商贸、旅游业优化发展，世界名牌折扣店签约进驻三水区，一批城市商圈设施不断完善。成功举办第二届佛山旅游文化节。文化创意产业、会展经济和总部经济发展迅速，佛山创意产业园初具规模，全国首个动漫主题虚拟创业园落户南海区，“国家工业设计与创意产业（顺德）基地”正式运营。

　　自主创新能力增强。我市荣获“创新型（2008）国家十强市”称号。佛山高新区实行“一区六园”新管理体制，佛山国家火炬创新创业园开工建设。产学研合作全面推进，公共研发服务平台建设加强。新增国家级检测中心2个、省级研究院1家、省级工程中心12家、院士工作室1个、博士后工作站10家。品牌、专利、标准战略实施取得新成绩，新增中国驰名商标6件，28个产品进入中国名牌产品公示阶段；参与制定并发布57项国家标准、13项行业标准；发明专利申请量和授权量均居全省前列。成功举办第五届中国国际专利与名牌博览会。

　　（二）以提升对外开放水平为目标，大力发展内外源型经济

　　民营企业发展环境优化。全市规模以上民营企业完成工业总产值6162.88亿元，增长21.9%。落实扶持民营企业的各项政策措施，加强对中小企业的服务，发挥政府参与设立中小企业信用担保基金的融资放大效应，推动全市担保行业累计为中小企业提供融资担保近180亿元。南海区实施民营企业发展“雄鹰计划”[2]，帮助企业解决发展中面临的各种难题。

　　招商选资力度加大。成功举办第二届佛山投资环境推介暨项目洽谈会，推出147个优质招商项目，签约投资239亿元。先后赴日韩、澳大利亚、东南亚、西班牙等国家和地区开展招商推介活动。全市实际外商直接投资18.07亿美元，增长14.9%。全年引进投资超千万美元项目66个，新增世界500强项目13个。

　　外经贸工作有效开展。积极应对国际金融危机，推动对外贸易结构转型升级。出口产品结构和市场结构进一步优化，机电产品和高新技术产品均保持增长，中东、印度、俄罗斯、拉美、非洲等新兴市场出口增势良好。全市外贸进出口总值422.14亿美元，增长11.6%，其中出口289.6亿美元，增长10.6%。鼓励企业参与联合国采购，截至去年底，全市共有587家企业成为联合国采购注册供应商。企业“走出去”取得实效，新批准设立境外企业16家。

　　（三）以交通基础设施建设为先导，大力推进现代化大城市建设与管理

　　以广佛轨道交通为重点的路网建设扎实推进。广佛轨道交通佛山段建设稳步推进，与广州主干路网的衔接有序进行。广佛-佛开高速扩建、广贺高速（三水段）、广明高速（西樵至更楼段）、江肇高速（高明段）等9个项目加快建设，广明高速二期、广三高速扩建等7个项目抓紧前期工作。广珠城际轨道交通建设顺利推进，途经我市的铁路工程线行选址、征地拆迁等前期工作进展顺利。启动“佛山一环”南拓工程建设，一批市重点路桥项目建设加快推进。航道整治和港口建设力度加大，东平水道创建成为省文明样板航道。

　　公共交通加快发展。公交优先发展政策进一步落实，《佛山市公共交通发展十大措施》得到实施。禅城区率先引入公共交通共同体管理新模式（TC）[3]，南海区桂城街道、顺德区北滘镇率先开通镇内公交，市民出行环境不断改善。

　　组团城市建设稳步推进。东平新城“南延东拓”发展范围初步确定，一批在建项目及配套基础设施建设进展顺利。大良-容桂组团供水管网连通工程建设任务完成。其他城市组团建设步伐加快。

　　“三旧”改造全面展开。完善“三旧”改造控制性详细规划和修建性详细规划，出台鼓励支持“三旧”改造的政策措施，改造过程中的一些问题得到解决。全市启动“三旧”改造项目209个，完成改造项目69个，完成改造面积632.77万平方米。“岭南天地”、中国家居博览城等大型改造项目开工建设。

　　城市基础设施建设步伐加快。电源建设和电网改造不断加强，天然气站和高压管网建设加快推进。第二水源地建设进展顺利。三年城乡防灾减灾工程累计完成建设总任务的92.4%。启动“无线佛山”建设，全市信息化综合指数达76.8％。

　　城市综合管理继续加强。理顺城市管理体制，形成市、区、镇（街道）、社区四级城市管理格局。强化城市属地管理职能，探索“联合执法、专群结合、综合治理”城管新模式，城市综合执法和综合管理力度加大，城市环境不断改善。

　　（四）以汾江河综合整治为抓手，大力加强节能减排与环境保护

　　节能减排强势推进。通过加大对污染行业的整治、推进环保重点工程建设和实施产业结构调整，节能减排取得新成绩。违法违规用地专项整治全面展开，全市复耕复绿土地2.22万亩，拆除违章建筑186万平方米。

　　水和大气环境综合整治力度加大。大力推进重点污染河段整治，全市累计完成主干河涌整治427.6公里，完成投资9.92亿元。实施汾江河综合整治“310”工程[4]，完成工程项目11项。加强污水处理厂及配套管网建设，新建、扩建污水处理厂4间，新增污水处理能力9.3万吨/日。大力推进大气污染防治，企业脱硫、使用清洁能源和“上大压小”工程建设不断加强，南庄、大沥和罗村等重点区域烟尘治理成效明显。

　　“绿地佛山”建设取得实效。确定“不开发区”战略。大力推进旧城区、道路的绿化提升和“示范路”建设，新增绿化面积727万平方米，改造绿化面积995万平方米，建设区级示范路3条、镇级示范路29条，“佛山一环”绿化提升工程任务全面完成。深入开展全民植树造林活动，全年植树311.4万株，营造各种主题林122个，完成山上造林3.92万亩。

　　（五）以改革创新为动力，大力推进区域协调发展

　　体制机制改革力度加大。行政管理体制改革继续深化，启动新一轮行政审批制度改革，审批效率大幅度提高；三水区镇（街道）区划顺利调整，市直交通综合行政执法改革全面完成，佛山高新区管委会管理体制实现创新。事业单位聘用制度基本建立，分类改革全面启动。农村体制改革稳步推进，制定《关于深化农村集体经济管理体制改革试点工作意见》，南海区出台政策有效解决农村“外嫁女”及其子女权益问题，全市引入中介组织规范农村财务管理工作基本完成。财政体制改革积极推进，公共财政继续向民生倾斜，全年财政投入公共服务和民生事业201.47亿元，占一般预算支出的82.5%，增长20.1%。试行了专项资金支出封套定额包干管理办法，开展了财政支出绩效评价改革。投融资体制改革扎实推进，出台《佛山市政府投资非经营性项目代建制管理办法（试行）》，成功举办2008佛山经营城市论坛暨城市可经营项目签约仪式，签约83项，投资总额442.26亿元。农信社改革顺利推进。信用佛山建设持续加强，我市被评为第三批中国金融生态市。

　　区域合作与发展积极推进。从政策措施、资金、项目和人力资源等方面加强对各区的支持，千方百计统筹推动各区协调发展、加快发展。以交通路网建设为切入点，加强与广州的合作发展，“广佛城巴”、“广佛快巴”如期开通，广佛年票实现互认互通。与港澳在金融服务、现代物流等方面的交流合作取得新成果；与国内外友好城市的文化交流、经贸合作进一步加强。

　　（六）以改善民生为重点，大力发展社会事业

　　社会建设取得新成绩。就业和社会保障工作不断加强，贯彻落实《劳动合同法》，在岗职工劳动合同签订率达96.7%。四级就业服务体系进一步完善，全年安置失业人员4.24万人，介绍各类求职者就业22万人。社会保险待遇调整工作得到落实，征缴面进一步扩大，综合征缴率达98%。教育事业均衡发展，全市新增省、市一级幼儿园31所。职业技术教育整合、转型及新校园建设步伐加快，教育现代化建设试点工作稳步推进。文化体育事业蓬勃发展，公共文化服务体系不断完善，文化遗产保护工作继续加强，祖庙修缮工程顺利推进。“敢为人先、崇文务实、通济和谐”成为新时期佛山人精神。“魅力佛山”、“全民健身与奥运同行”、“市民自行车大巡游”等系列文体活动取得成效。卫生和人口事业加快发展，三级卫生服务网络进一步完善，全市共设置社区卫生服务中心45个、服务站230个。免费婚检、产检和出生缺陷干预工程积极推进，人口计生综合改革力度加大，我市户籍人口政策生育率96.2％，人口自然增长率4.81％。。全力支援汶川地震灾区抗震救灾和开展对口援建，累计捐款4.13亿元，救治、转送伤病员4638人，完成1.13万套22.5万平方米过渡安置房生产任务。

　　平安佛山创建不断深化。推进“六好”平安和谐社区、“十好”和谐文明村居创建活动取得新成果。加强严打整治和综合治理，强化国家安全工作，社会大局总体稳定。开展领导“大接访”活动,及时解决了一批群众反映的实际问题。成功应对自然灾害、手足口病、“三聚氰胺”等突发事件。开展产品质量、农产品安全检测和食品安全专项整治工作，强化安全生产监管，公共安全水平不断提高。供应北京奥运会食品安全保障任务圆满完成。三水区成为我省首批国家级食品安全示范区。

　　十件民生实事顺利完成。去年《政府工作报告》确定的十件民生实事顺利完成。市级财政安排5000万元、各区配套资金2.3亿元支持旧村居改造。各级财政投入8715万元实施渔民上岸工程，解决无房户渔民住房720套。建设廉租房2068套。非户籍常住人口子女中的13类政策性借读生及华侨子女、台湾学生已经纳入免费义务教育对象范围。先后建立居民门诊基本医疗和职工门诊基本医疗制度，在全省乃至全国率先步入全民医保时代，全市参保人数362.5万人；全市二级以上医院设立“惠民平价门诊号”，新增提供“惠民产房”服务定点医疗保健机构11家。免费培训失业人员8880名、高技能人才9127名、本地农村富余劳动力1.21万名。企业退休人员月人均基本养老金提高到1070元。调整低保标准，禅城、南海、顺德区调整为320元/人·月，高明、三水区调整为280元/人·月。为1.5万人次适龄低保家庭子女落实义务教育阶段生活补助政策。对全市低保家庭实行燃气价格补贴，食品消费增支部分按月给予补贴。

　　（七）以“廉政效能服务”为主题，大力加强政府自身建设

　　民主法制建设得到加强。坚持向人大报告工作和向政协通报工作制度，自觉接受人大和政协的监督。认真办理人大代表议案、建议和政协提案，办复率100%；认真听取各民主党派、工商联、无党派人士、人民团体、社会组织和市政府顾问意见，科学民主决策水平进一步提高。政府法制建设和政务、厂务、村务公开取得成效，普法、执法、行政监察和审计监督工作有效开展。

　　解放思想活动取得新成果。全面开展解放思想学习讨论活动，明确以“现代制造基地、产业服务中心、岭南文化名城、美丽富裕家园”为佛山发展定位。先后提出并实施金融发展三项计划、信息化发展“3＋1”规划[5]，出台加快推进产业强市战略的若干意见及14个配套文件、促进经济平稳较快发展十大措施等，政府引领经济社会发展的能力不断增强。

　　政风和廉政建设进一步加强。开展以“廉政效能服务”为主题的机关作风建设年活动，机关作风建设不断加强。启动行政投诉电子监察系统，充分发挥“12345”行政服务、行政投诉热线作用，查处并纠正严重影响行政效能行为。认真落实党风廉政建设责任制，严肃查处了一批违法违纪案件。加强公务员队伍能力建设，政府服务效率和水平不断提高。

　　与此同时，老年人、残疾人、妇女儿童事业，粮食储备、民族宗教、外事侨务、对台港澳事务、广播电视、新闻出版、统计、档案、方志、人防、气象、扶贫等各项工作都取得了新成绩。

　　各位代表！一年来我们取得的成绩来之不易。这是省委、省政府和市委正确领导的结果；是全市人民抢抓机遇，克服困难，开拓进取，团结奋斗的结果；是各位人大代表、政协委员和社会各界人士监督支持的结果。在此，我代表佛山市人民政府向全市广大干部群众，中央、省驻佛山单位，驻佛山人民解放军指战员、武警官兵，人民警察和社会各界人士表示崇高的敬意，向长期关心支持佛山建设的港澳台同胞、海外侨胞、国内外友人以及外来务工人员表示衷心的感谢！

　　我们也清醒地认识到，我市经济社会发展中仍然存在一些矛盾和问题。主要是：产业结构调整优化任务艰巨，在调整中科学把握好二三产业的良性互动和均衡发展难度很大；各种资源要素约束刚性增强，经济发展空间越来越受到限制；自主创新动力机制亟待加强；节能减排和生态环境保护任务依然严峻；统筹解决“三农”问题压力较大，区域发展不够平衡；食品药品安全、安全生产及维护社会稳定等涉及人民群众切身利益的突出问题需要进一步解决；政府管理和公共服务能力有待进一步提高，等等。我们要正视这些矛盾和问题，并在今后工作中认真加以解决。

　　二、2009年面临的形势和目标任务

　　2009年，是新中国成立60周年，是我市推进经济社会全面转入科学发展轨道，实现“两转型一再造”目标的重要一年，也是实施《珠江三角洲地区改革发展规划纲要》的开局之年。做好今年的工作，意义重大。

　　综观国际国内经济形势，2009年将是新世纪以来我市经济社会发展最为困难的一年，国际金融危机尚未见底，严重后果还将进一步显现。国内经济运行困难增多，经济下行压力增大，企业经营普遍困难，市场消费信心不足。受国际金融危机影响，我市经济增长速度将会放缓，促进经济增长的任务艰巨。但危机中蕴藏着重大发展机遇，我们看到，我市今年发展也面临许多有利条件。一是我国经济发展的基本面和长期趋势没有改变。为促进经济增长，中央提出了积极的财政政策和适度宽松的货币政策，出台了一系列扩内需、保增长、调结构的政策措施，强调把保持经济平稳较快发展作为当前的首要任务，显示了中央促进经济增长的信心和决心。二是《珠江三角洲地区改革发展规划纲要》的出台，明确了珠三角地区在全国改革发展大局的战略定位，必将有力地促进佛山经济发展、城市建设、文化提升、民生改善实现新跨越。三是我市有30年改革开放积累的雄厚的经济基础、完善的基础设施、完备的产业配套、较高的市场化水平，加上我市以制造业为主、以民营企业为主、规模企业发展迅猛，等等，为我市应对国际金融危机创造了有利条件。四是我们对问题认识较早，应对举措及时有效，出台了促进经济平稳较快发展十大措施，特别是先后与国内主要商业银行签订了总额达2800亿元的授信协议，有力地缓解了我市建设和发展中的资金问题。我们认为，只要我们正确估计形势，客观分析影响，勇于面对挑战，抢抓发展机遇，尤其要紧扣“科学发展、先行先试”这条主线，从解决最紧迫、最突出、最重大的问题入手，扎实推进规划纲要的实施，全面落实中央、省、市促进经济增长的系列政策措施，佛山一定能克服困难，实现率先发展、科学发展！

　　2009年政府工作的总体要求是：以邓小平理论和“三个代表”重要思想为指导，深入贯彻科学发展观，全面贯彻党的十七大、十七届三中全会、中央经济工作会议和省委十届四次全会精神，贯彻实施《珠江三角洲地区改革发展规划纲要》，按照市委十届六次全会的总体部署，以建设“现代制造基地、产业服务中心、岭南文化名城、美丽富裕家园”为目标，把保持经济平稳较快发展作为首要任务，把扩大内需作为保增长的根本途径，继续解放思想，坚持改革开放，加快发展方式转变和结构调整，加快自主创新和体制机制改革，加快城乡经济社会一体化发展，加快改善民生和促进社会和谐，努力把佛山建设成为广东参与国际竞争的先锋队，推进珠三角一体化的主力军，探索科学发展模式的排头兵。

　　2009年经济社会发展主要预期目标是：生产总值增长11%；人均生产总值力争与本地生产总值同步增长；单位GDP能耗下降4.36%；主要污染物二氧化硫和化学需氧量排放量分别下降6%和5%；全社会固定资产投资增长12%；社会消费品零售总额增长16%；地方财政一般预算收入增长9%；进出口总值力争有所增长；城市居民人均可支配收入增长7.5%；农村居民人均纯收入增长7%；居民消费价格总水平涨幅控制在4%以内；城镇登记失业率控制在4%以内；人口自然增长率控制在6.3％。以内。

　　实现今年经济社会发展目标任务，重点要抓好“三个着力”：

　　（一）着力推进产业发展转型

　　做精做优做强现代农业，提升农业综合生产能力。以都市农业、生态农业、观光农业、外汇农业和品牌农业为方向，推动传统农业向现代农业转型。抓好陈村花卉世界、国通物流城等重点项目建设，发展壮大一批农业龙头企业。完善农产品交易市场和流通网络，加快形成生产、加工、流通有机结合的产业链，提升农业产业化、组织化水平。大力发展农民专业合作社，完善多层次、多功能的农业社会化服务体系。加强农业基础设施建设，高标准建设现代农业生态园区和海峡两岸农业合作试验区。加强优势农产品科技推广示范基地建设，推进农业科技创新和成果转化，提高科技对农业的贡献率。实施信息兴农工程，以信息化促进农业产业化。推行农业标准化及无公害生产，加强有害生物防控工作，提升农产品消费安全水平。

　　调整优化产业结构，建设现代产业体系。坚持先进制造业和现代服务业相配套，信息化与工业化相融合，调整优化产业结构，提升产业国际竞争力。大力发展先进制造业，重点发展装备制造、汽车及零配件、新型显示器件等产业，优先发展电子信息、生物制药、医疗器械、新能源、新材料、环保等新兴产业和高新技术产业，继续用高新技术、先进适用技术和现代管理技术改造提升家电、建材、纺织等优势传统产业。加快建设“3+9”特色产业基地[6]，大力推进广东省无线射频产业（佛山）基地、广东薄膜太阳能产业基地和顺德国家光伏系统工程研究中心产业化基地等建设，促进加快形成具有佛山特色的现代产业体系。实施信息化发展“3＋1”规划，以信息化带动工业化，加快信息技术与工业设计、产品构成、生产过程、经营管理等方面融合，全面提升产业优势。以招商引资促进产业结构优化升级，把欧美、日韩、新加坡、台湾等国家和地区作为招商重点区域，着力引进先进制造业、高新技术产业和现代服务业。办好第三届佛山投资环境推介暨项目洽谈会。扶持发展一批具有国际竞争力的大企业集团和产业集群。推进产业集群建设与资本运作有机结合，争取成为联合国产业集群与资本运作试点城市。积极实施“双转移”战略，按照省示范性产业转移园标准协助建设佛山（清远）、（云浮）产业转移园，配合做好园区招商引资和项目建设。加快发展现代服务业，重点发展金融业、物流业、会展业、文化创意产业和总部经济等。抓紧落实扶持促进广东金融高新技术服务区建设的政策措施，加大招商力度，今年力争引入多家银行机构。全面实施金融发展三项计划，今年要在推动设立科技创业孵化基金，试点非标准公司股权质押融资等创新项目上取得突破。推进中新（佛山）现代物流工业园、华南国际采购及物流配送中心等重大项目建设。结合产业优势和特点，整合各类会展资源，打造一批知名会展品牌。完善总部经济发展规划，建设一批总部经济大楼，形成总部经济带。大力发展以工业设计、网游动漫等为基础的创意产业，加快建设创意产业园区，推动“佛山制造”向“佛山创造”转变。优化发展商贸旅游业，推进世界名牌商品折扣店、大型商品零售批发机构、云东海等建设。办好第三届佛山旅游文化节。做好第二次经济普查工作。

　　加快内外源型经济“双轮驱动”，提升经济国际化水平。贯彻落实国家和省扶持中小企业的各项优惠政策，推进“减税、让利、减费”工作，切实减轻企业负担。引导中小企业找准发展定位，及时调整市场策略和产品结构，加大内销市场开发力度。鼓励和支持企业参与“家电下乡”，拓展农村消费市场。加强和改善政府服务，帮助企业解决发展中面临的资金、土地、人才等实际难题。引导和鼓励中小企业进行股份制改造，推动建立现代企业制度。积极促进外经贸稳定发展，进一步优化出口产品和市场结构，扩大高新技术产品、具有自主知识产权产品的出口。支持行业协会和企业组团参加国际展览、展销及境外经贸活动，通过展会平台扩大出口。积极开拓俄罗斯、东盟、中东、南美洲、非洲等新兴市场。以信息化加快国际化，加强国际贸易电子商务平台建设，鼓励企业利用阿里巴巴等知名电子交易平台拓展国内外贸易。大力推进加工贸易转型升级，鼓励和支持加工贸易企业开拓内销市场，扩大国内采购及产品内销。继续推动企业参与联合国采购。加快“走出去”步伐，鼓励和支持企业到国外投资设厂，开展国际化经营。积极应对国际贸易壁垒和摩擦，改进口岸通关服务，创造良好外贸环境。

　　提高自主创新能力，建设创新型佛山。围绕建设现代产业的需要，着力抓好关键领域引进消化吸收再创新和集成创新，加快创新成果转化。以新能源技术、新型显示技术、LED（发光二极管）等为突破口,集中力量解决一批关键领域、核心技术和行业共性技术。继续深化产学研合作，促使一批重点院校、科研院所与我市共建产学研技术创新联盟。以专利、标准和名牌为切入点，继续推进国家知识产权示范城市建设，鼓励企业参与技术标准体系的研制；扶持发展一批具有国际影响力的名牌产品和区域品牌。加快建设一批重点实验室、检测认证中心等公共技术平台和创新服务体系，突出抓好佛山国家火炬创新创业园建设，全力打造国家级孵化器。构建社会化、多元化科技创新投入体系，用好科技发展专项基金。发挥政府采购扶持自主创新的作用。实施人才开发战略，完善和落实人才政策，加快建设一支高层次、国际化的优秀人才队伍，重点建设博士后工作站、留学人员创业园。

　　坚持统筹协调、分类指导，促进五区共同发展。支持五区按照城市发展定位，充分发挥各自特色和优势，实行差异化发展。进一步加强统筹力度，对重大项目在全市五区统筹安排；进一步完善规划实施机制，优化各城市组团功能布局，推进城市建设和重大基础设施、公共服务设施的衔接；进一步简政放权，发挥区、镇（街道）在推动区域经济发展的积极性、创新性。加快制定科学发展考核评价指标体系，引领五区科学发展、协调发展。统筹全市资源，支持禅城区大力发展城市精品经济，提升中心城区综合竞争力；建设“岭南天地”。支持南海区加快建设广东金融高新技术服务区；打造先进制造业基地。支持顺德区推进综合体制改革试点工作；加快构建具有国际竞争力的现代产业体系。支持高明、三水区加快重大项目和民生事业建设，努力实现科学发展、跨越发展。

　　（二）着力推进城市发展转型

　　加强城乡规划统筹，推进城乡一体化发展。抓住《珠江三角洲地区改革发展规划纲要》实施带来新一轮城镇化发展的机遇，加强城乡规划统筹，将土地利用、城乡空间布局、产业发展、基础设施建设、生态环保、公共服务等规划作为一个整体统筹编制，完善城乡规划体系。今年要抓好镇（街道）、村（居）规划的修编完善，推动镇村规划与城市规划建设相衔接，使全市城乡真正实现规划对接、资源共享、联动发展。完善“不开发区”规划及实施机制。推进城乡公共设施均等化，加快农村道路交通、供水供电、广播电视、信息网络、生态环境等基础设施建设，促进城市公共设施向农村延伸，实现市、区、镇、村四级之间公共基础设施的对接。推进城乡公共服务均等化，加快农村科技、教育、文化、卫生、体育等社会事业发展，促进金融、职业培训、就业和社会保障等公共服务向农村延伸覆盖，引导城市资本、科技、人才为农村提供社会服务。

　　建立强农惠农长效机制，采取有利于统筹城乡发展的税收政策和公共财政制度安排，扩大公共财政覆盖农村的范围，加大对“三农”投入力度，逐步缩小城乡差距。选择综合实力较强、基础条件较好、区域发展相对均衡的镇（街道），作为开展统筹城乡发展综合改革试点，以点带面，逐步带动全市城乡一体化发展。

　　加强土地科学使用，塑造城乡宜居宜商土地利用空间。严格落实土地利用总体规划确定的耕地保有量、基本农田面积、城乡建设用地总量等各项指标。把握我市作为广东省推进节约集约用地试点示范市的机遇，积极探索土地利用新模式，力争在优化用地结构布局、开拓土地利用途径、确保重点发展用地等方面实现新突破。实施“三旧”改造“双百计划”[7]，最大限度地盘活使用土地存量。协调促进城乡建设规划、综合交通规划与土地利用总体规划的衔接，强化规划调控土地的主导作用。加强对土地投资强度和投入产出率等指标考核，提高土地利用效率。严禁违法违规用地，健全依法依规用地长效机制，全面落实政府主导、国土部门牵头、其他部门配合的土地管理共同责任制。完成新一轮土地利用总体规划修编和全市第二次土地调查工作。

　　加强基础设施建设，提升城市综合竞争力。全面实施《佛山市综合交通规划（2008-2020年）》。加快与广州新白云机场、新火车站、南沙港等三大交通枢纽的路网对接，继续推进与广州、中山、江门、肇庆等周边城市主干道路的衔接，抓紧做好与港珠澳大桥道路规划的衔接，完善我市与周边城市的路网体系。稳步推进广佛轨道交通建设，启动广佛南延线建设，力争广佛环线立项动工。加快“佛山一环”南拓工程和市重点路桥项目的建设步伐，完善全市区域区与区之间的路网体系。配合做好途经我市的广珠城际轨道交通、武广、贵广、南广、广茂等铁路及珠二环、广珠西线等高速公路建设。抓紧编制佛山西站、新三水站和丹灶铁路物流中心、官窑货运站的建设规划。做好佛山机场民航复航筹备工作。加大港口、码头、河道等资源整合和建设力度，力争东平水道创建为国家文明样板航道。落实公交优先发展政策，加强公交站场和轨道交通接驳站场的规划建设，进一步优化完善全市公交网络，大力发展镇内公交，继续推进广佛客运公交化、同城化，为市民出行提供更便捷服务。采取更强有力措施推进电源电网和“上大压小”工程建设，缓解我市电力供应难题；继续推进天然气站和高压管网建设。加快第二饮用水源工程建设，力争下半年实现试通水。抓好第二轮城乡防灾减灾工程建设。

　　加强组团城市建设与管理，提升城市现代化水平。进一步完善组团城市规划及实施机制，统筹全市资源加快推进组团城市建设。东平新城要加快推进公共文化综合体[8]、公共服务中心、交通枢纽中心等项目建设，继续完善水利、道路、绿化等配套基础设施建设。大良-容桂组团要加大建设力度，尽快形成百万人口中心城区。西江新城、西南组团等其他城市组团要加快建设步伐，推动城市组团之间有效衔接与错位发展。以信息化提升城市化，加快电信网、互联网和有线电视网“三网融合”，打造“无线城市群”，建设“U-佛山”[9]。加强信息基础设施建设，重点建设全市统一的电子政务网络数据中心、应急指挥中心、灾难备份中心。加快“数字化城管”建设，优先在市及各区中心城区建设数字城管系统网络，力争用三年左右时间，建成覆盖全市的“网格化城市管理”模式。完善城市综合执法和综合管理机制，突出抓好违法用地、违法建设的查控及市容市貌的整治，努力建设一个安定有序、文明洁净的现代化城市。

　　全面落实《珠江三角洲地区改革发展规划纲要》，促进区域经济一体化。把握珠三角地区改革发展规划上升为国家战略的重大机遇，以推进广佛同城化为突破口，主动与广州密切联系，共同编制“广佛同城化”建设战略规划，就产业、交通、金融、科技、教育、医疗、文化旅游、生态环境、就业和社会保障等方面开展深入合作，率先构建城市规划统筹协调、基础设施共建共享、产业发展合作共赢、公共事务协作管理的一体化发展格局；以交通基础设施一体化为切入点，积极加强与周边城市在交通路网、资源配置、生态环保、科技创新、市场体系、信息化建设等方面的规划衔接和协作联动，突破行政体制障碍，共同推进珠三角区域经济一体化。抓住我市成为商务部首批落实CEPA示范城市和广东省CEPA 5先行先试重点市的机遇，以项目为纽带，积极推进与港澳在金融服务、现代物流、电子商务、会展、科技等方面合作。支持我市企业到香港上市融资。力争我市成为粤港澳金融合作区的金融创新试点城市。继续加强与国内外友好城市的交流合作，推动交流合作向更宽领域、更深层次、更高水平发展。

　　（三）着力推进环境再造

　　推进生态环境建设，提升城市发展集聚力。严格执行主要污染物排放总量控制制度，建立污染减排倒逼机制。加大工程减排、结构减排和监管减排力度，全力抓好截污工程、污水处理厂及配套污水收集系统、引水泵站等工程建设，力争新建、续建、扩建24间污水处理厂，新增污水处理规模约75.5万吨/日，城镇污水处理率达到77%。继续加强对传统耗能企业的整治提升，健全落后产能有效退出机制，坚决淘汰一批落后产能。大力推广节能新技术，加快形成节约型生产和消费模式。大力发展循环经济，综合运用市场、价格、信贷、环保等手段，引导推动企业和重点行业开展清洁生产、节能降耗和资源综合利用。继续推进以汾江河（佛山水道）、内河涌综合整治为重点的水环境治理，完成综合整治各项任务，稳步改善主要河涌水质。继续推进以改善空气质量为重点的大气污染防治，加强电厂、工业锅炉、窑炉等企业脱硫工程建设和清洁能源改造，着力治理机动车尾气、施工扬尘、餐饮业油烟，不断改善空气环境质量。提升生活垃圾无害化处理能力和管理水平，大力推行垃圾分类收集和处理，最大限度地实现生活垃圾减量化目标。完善在线监控系统，强化环境监督执法，严厉打击环境违法行为。全面实施“美丽佛山”战略，继续推进立体绿化，突出抓好道路、河涌、庭院绿化和村后风景林改造，加快构建全市道路绿网骨架及河道绿网系统。大力开展全民植树造林活动。积极推进国家级生态示范镇（村）、全国环境优美乡镇等创建活动。做好迎接国家园林城市复查工作。

　　继续深化体制机制改革，增强促进科学发展的动力和活力。积极推进农村体制改革，利用我市作为广东省开展统筹城乡发展综合改革试点市的优势，按照“培育、整合、改造、规范”四种类型，探索农村集体经济管理体制改革，今年重点抓好村居改革试点工作。推动和规范土地承包经营权流转，逐步建立城乡统一的建设用地市场。完善征地补偿机制，解决好被征地农村居民就业、住房和社会保障。进一步理顺农村基层党组织、自治组织与经济组织的关系。稳步推进行政管理体制改革，以顺德区为试点，制订并实施“大部制”改革方案，推进政府机构及其职能调整，完善政府组织体系和行政运行协调机制。继续推进行政审批制度改革，加强全市各级行政服务中心的统筹规划和统一管理，加快行政审批电子网络一体化建设，努力提高政府服务效能。加快推进事业单位分类改革。进一步推进国有企业改革，抓好劣势企业退出清理工作，推进水业、气业上市和上市公司重组，加强国有资产监管，促进国有资产增值保值。加大财政体制改革力度，加强税收征管，规范非税收入管理。调整优化财政支出结构，大力节约财政开支和压减一般性财政支出，扩大财政对公共事业、民生事业和“三农”工作的投入，落实财政对中小企业的扶持政策。积极推进绩效预算、财务集中监管、公务卡结算制度等改革，确保财政资金安全高效、用到实处。深化金融体制改革，建立完善再担保机制和体系，发展创业风险投资基金、创业孵化基金等，为中小企业提供优质融资服务。积极开展资本运作，抓住创业板即将推出的有利时机，引导、鼓励一批优质民营企业尽早进入。建立健全金融风险预警机制和管理体系，增强防范化解金融风险能力。优化金融生态环境，建设“信用佛山”。完善投资体制改革，进一步规范政府投资项目管理，完善招标、投标行政监管机制。办好2009佛山城市可经营项目投资推介洽谈会。大力促进行业协会、商会和社区民间组织等社会组织发展，建立健全政府对社会组织的培育和扶持机制，改革完善社会组织的监管模式，引导社会组织诚信自律，提高社会组织工作人员职业素质和专业水平。

　　推进人文环境建设，提升城市发展核心力。加强社会主义精神文明建设，大力弘扬社会主义核心价值和新时期佛山人精神，营造共享和谐、积极向上的社会环境。以创建全国文明城市为抓手，广泛深入开展社会公德、职业道德、家庭美德、个人品德教育，进一步提升市民文明素质和城市文明程度。加强国防动员和民兵预备役建设，进一步提高双拥工作水平。推动文化创新，不断提升佛山的文化软实力。完善城乡公共文化服务体系，加强基层公共文化设施建设，推动全市文化资源共享。做大做强“魅力佛山”文化品牌，大力开展周末系列、流动系列和高雅艺术系列文化活动，加强基层和谐文化建设，丰富广大市民和外来务工人员文化生活。加强体现岭南特色文化遗产的保护开发利用，重点抓好祖庙-东华里历史文化街区、南风古灶片区改造，基本完成祖庙修缮工程。利用我市陶艺、粤剧、武术等传统文化优势，打造具有影响力的佛山特色旅游文化品牌。大力实施《佛山市文化产业发展规划（2008-2020年）》，运用信息技术创新文化产业发展方式，促进文化产业不断发展壮大。加快体育事业发展，推动公共体育设施完善和共享。以迎亚运为契机，办好佛山市第七届运动会和亚洲游泳锦标赛。

　　推进政务环境再造，努力建设廉洁勤政务实高效政府。大力加强法治政府建设，自觉接受市人大的法律监督和工作监督，积极支持市政协的民主监督，主动听取各民主党派、工商联、无党派人士、人民团体和社会各界的意见和建议，重视群众监督和舆论监督。认真办好人大代表议案、建议和政协提案。进一步完善政府决策机制，坚持政府重要事项和公共政策社会公示制度，完善行政决策专家论证、社会听证和合法性审查制度，积极推进政务公开和“阳光工程”。进一步创新政府管理，积极推动理论创新、制度创新、管理创新和服务创新，不断提高引领科学发展能力。进一步转变政府职能，完善政府组织体系，加强条块之间的合作和部门之间的沟通，确保政令畅通，切实提高政府执行力。推行行政首长问责制，加强行政效能监察和行政执法监督，在政府重点工作分解落实基础上，建立健全重大决策执行信息反馈、目标管理和绩效考评机制。加强反腐倡廉制度建设，做到用制度管权、管事、管人，加快形成政府反腐倡廉制度体系和权力运行监控机制。全面落实党风廉政建设责任制，完善领导干部廉洁自律和民主监督制度。扎实开展各专项整治工作，严肃查处各种腐败行为。继续深入开展机关作风建设活动，努力塑造务实创新、勤政为民、廉洁高效的政府形象。

　　加快社会事业发展，构建稳定和谐发展环境。加强就业和社会保障工作，完善城乡一体化培训就业服务体系，建立健全城乡一体化人力资源市场。鼓励企业不实行生产性裁员。实施以创业带动就业工程，开展城乡再就业援助活动。推广顺德区农民创业园建设经验，为就业困难群众提供就业渠道。积极做好高校毕业生就业工作。以顺德区为试点，研究出台全征地农村居民养老保险实施方案。扩大工伤保险覆盖面，重点推动建筑企业农民工参加工伤保险。创新具有佛山特色的企业退休人员社会化管理服务模式。完善社会救助体系，落实低保和“五保”供养工作。实施低收入居民临时生活补助与物价涨幅联动，缓解物价上涨对低收入居民生活的影响。优先发展教育事业，继续推进教育现代化建设试点工作。加快义务教育规范化学校建设，促进义务教育均衡发展。加快佛山科学技术学院和佛山职业技术学院新校园建设，推动顺德职业技术学院进一步形成办学特色。积极稳妥解决中小学代课教师问题。加强国际教育交流，推进教育国际化进程。加强与东西两翼和粤北山区合作办学，完成省下达我市的培训任务。大力发展卫生事业，加强公共卫生和医疗服务体系建设，有效提高突发公共卫生事件应急处理能力。继续完善“惠民门诊”、“惠民产房”服务，规范门诊基本医疗保险服务流程。改进医德医风，建立和谐医患关系。强化医疗行业监管，健全医疗机构管理长效机制。广泛开展群众性爱国卫生运动。加强人口与计生服务管理工作，稳定低生育水平，提高出生人口素质。深入开展“六好”平安和谐社区、“十好”和谐文明村居创建活动。健全社会治安防控体系，强化社会治安综合治理，加强针对治安重点区域和“两抢一盗”等突出治安问题的严打整治，争取用三年左右时间实现社会治安明显好转。加强国家安全工作。强化安全生产企业法人主体责任和政府监管主体责任，深入开展各项安全生产专项整治行动，防范和遏制重特大事故发生。严格农产品、食品、药品质量监管，保障人民群众饮食和用药安全。完善自然灾害、环境污染、公共安全等突发事件应急机制。健全信访工作机制，积极妥善化解社会矛盾。加强流动人口和出租屋的管理与服务，维护流动人口合法权益。继续做好对口支援四川水磨镇恢复重建工作。

　　着力办好十件民生实事：

　　1、开展政策性农业保险试点工作。按照“政府引导、政策支持、市场运作、自愿参保”的模式，率先推出水稻保险试点工作，争取试点险种覆盖全行业达到40%以上。

　　2、加强保障性住房建设。落实住房保障建设责任，完成建设保障性住房2410套。继续实施渔民安居工程，全面解决264户人均住房面积不到12平方米的渔民住房问题。

　　3、加强基层医疗卫生机构建设。全面落实我市《关于进一步加强基层医疗卫生机构建设的意见》，新建45间社区卫生服务站,完成205间社区卫生服务站信息化基础设施配套。

　　4、完善居民基本医疗保险制度。提高居民住院和门诊基本医疗待遇；将大学生及中等职业技术学校学生纳入居民住院和门诊基本医疗保险范围，惠及学生8.47万多人。

　　5、加强农产品、食品安全监管工作。健全农产品质量安全监督检测体系。加大对屠宰厂（场）的规范整治力度，积极推进生猪来源定点基地供应，完善生猪协议准入、购销挂钩制度，逐步建立肉品安全追溯管理信息平台，确保我市生猪质量达到“放心肉”标准。

　　6、解决农村居民“出行难”问题。基本实现全市200人以上自然村、革命老区50人以上自然村通水泥（沥青）路目标，全面提升农村公路通行能力。

　　7、扶持革命老区建设。建立健全扶持老区工作机制，着力解决“一保五难”问题，改善老区生产生活环境。加大财政投入，今年优先解决老区“饮水难”问题。

　　8、改善市民文化生活质量。加快推进市图书馆、博物馆、艺术馆等新馆建设，为基层群众购买公益电影1.1万场，引进约15场国内外高水准的艺术演出和展览，扶持重点文艺社团开展创作、演出和对外交流活动，资助服务外来工的文化设施建设和文化活动。

　　9、继续提高城乡低保标准。禅城、南海、顺德区由320元/人·月提高到350元/人·月，高明、三水区由280元/人·月提高到310元/人·月。对城乡最低生活保障对象实施分类救助。

　　10、继续调整企业退休人员基本养老金。从今年1月起，对2008年12月31日前（含本日）已领取基本养老金的21.38万多名企业退休人员调整基本养老金。同时，妥善解决早期离开县以上国有集体企业人员社会保险问题。

　　各位代表！当前佛山正处于以新一轮思想大解放推动新一轮经济社会大发展的关键时期。推动科学发展、促进社会和谐的要求更加迫切，任务更加艰巨，责任更加重大。让我们在市委的坚强领导下，深入贯彻落实科学发展观，团结和带领全市人民，坚定信心，振奋精神，奋力拼搏，为实现“两转型一再造”目标，为建设我们更加美丽富裕的家园而努力奋斗！
